# Postgraduate General Practice Training Under Early Clinical Responsibility: A Narrative Review on System-Based Supervision and the Supportive Role of Artificial Intelligence

**DOI:** 10.3390/healthcare14040503

**Published:** 2026-02-15

**Authors:** Christian J. Wiedermann, Giuliano Piccoliori, Pietro Murali, Cristina Pizzini, Doris Hager von Strobele Prainsack

**Affiliations:** Institute of General Practice and Public Health, Claudiana—College of Health Professions, 39100 Bolzano, Italy

**Keywords:** general practice, family practice, education, medical, graduate, competency-based education, clinical supervision, primary health care, health care reform, artificial intelligence, clinical decision support systems, patient safety

## Abstract

**Highlights:**

**What are the main findings?**

- Traditional one-to-one apprenticeship models in postgraduate general practice are increasingly insufficient under the conditions of early clinical responsibility, multidisciplinary care, and primary care system reform.
- System-based supervision frameworks, supported by competency-based assessments and organizational accountability, provide a more scalable and resilient approach to postgraduate general practice education.

**What are the implications of the main findings?**

- Community Centers can function as effective learning organizations only if educational roles, supervisory responsibilities, and protected training capacities are explicitly integrated into primary care reform.
- Artificial intelligence can augment supervision and assessment in distributed training settings, but only within clearly governed systems that preserve human oversight, ethics, and professional accountability.

**Abstract:**

Background/Objectives: Primary care faces transformation due to workforce shortages and reform. Italy’s Decree 77/2022 promotes Community Centers and extended care, while postgraduate training in general practice involves early clinical responsibility. In South Tyrol, trainees assume significant patient care duties early in a three-year program. This review examines traditional apprenticeship-based training and explores system-based supervision and AI as strategies for improving quality and safety. Methods: A narrative review synthesized the literature and policy on postgraduate general practice education, supervised autonomy, and AI tools in primary care. Searches used the PubMed and Consensus platforms, focusing on Italian primary care reform and South Tyrol. Evidence was analyzed using SANRA guidance. Results: Evidence consistently indicates that training quality depends less on individual supervisors and more on structured, system-based supervision frameworks, clear entrustment criteria, and supportive organizational contexts. Early supervised clinical autonomy in community-based primary care settings can accelerate competency development without compromising the quality of care when robust supervision and team structures are in place. AI-supported educational tools have the potential to augment feedback, assessment, and learning analytics, especially in settings with limited supervisory capacity; however, current evidence supports their use only as adjuncts to human supervision. Conclusions: Evidence supports system-based, competency-oriented supervision models over traditional apprenticeships in settings characterized by workforce constraints and distributed training sites. Integrated general-practitioner-led primary care settings offer favorable learning environments for postgraduate training, while service-oriented community hubs need careful governance as training sites. Though AI may support supervision, professional oversight remains essential for quality and safety.

## 1. Introduction

Primary care is the cornerstone of an effective health system. Its performance depends on organizational structure, financing, and healthcare education quality. Patient complexity, multimorbidity, demographic changes, and expectations for accessible care have increased the demand for primary care services and professional competencies [1,2]. These pressures have exposed the limitations of traditional postgraduate education models for general practice.

Traditionally, postgraduate general practice training has used an apprenticeship model with one-to-one supervision by experienced practitioners. While valued for role modeling and continuity, evidence suggests that this model inadequately addresses modern team-based primary care needs [1,3,4]. Variation in trainer expertise and limited scalability under workforce constraints challenge the ability of these models to maintain educational quality [5,6].

These challenges are particularly salient in Italy, where postgraduate training in general practice has evolved under conditions of persistent shortages in the primary care workforce and marked regional heterogeneity [7,8]. Unlike systems in which prolonged protected training precedes independent clinical responsibility, Italian trainees frequently assume substantial patient care responsibilities early in their postgraduate training, often under their own professional accountability [8]. While Italian postgraduate GP trainees are not granted full independent legal responsibility as fully qualified general practitioners, current regulations and contractual provisions allow trainees to engage in compensated clinical roles, including provisional primary care assignments, during training [9]. In South Tyrol, general practice trainees may manage patient lists during their three-year program, supported by tutors rather than through constant supervision [10].

In parallel with these workforce-driven educational adaptations, primary care in Italy has entered a phase of profound structural reform. Ministerial Decree (DM) 77/2022 introduced a comprehensive reorganization of territorial healthcare, emphasizing Community Centers (Case della Comunità, CdCs), extended service availability, multidisciplinary teamwork, and digital health integration. The reform aims to strengthen primary care accessibility, continuity, and coordination while shifting care away from hospital-centered models [7,11]. However, the extent to which these objectives are realized depends on implementation models, particularly regarding the preservation of longitudinal, general practice-led care. Importantly, these reforms directly affect the settings in which postgraduate education occurs, creating new learning environments that fundamentally differ from traditional single-physician practices.

International reviews suggest that community-based multidisciplinary primary care settings enhance practice readiness and clinical competence when educational structures are well designed [12,13,14]. Early supervised autonomy accelerates competency development when clear entrustment criteria and supervision are established [15,16,17].

The convergence of early clinical responsibility and workforce shortages creates tension between service provision and educational quality. Concerns exist that service demands on trainees may reduce learning time and supervision quality, particularly in community settings. Evidence shows that training quality depends on system features such as organizational support, protected time, and governance structures [1,2,18].

Attention has been paid to system-based supervision models and AI-supported tools in postgraduate healthcare education. While evidence does not support replacing human supervision, AI-assisted feedback and analytics may help standardize education and enhance assessments, particularly in resource-constrained settings [19,20,21]. Ethical and legal considerations underscore the need for AI to augment, rather than replace, supervision.

Across European training systems, early clinical responsibility is most embedded within a sequential training structure, in which substantial hospital-based training under specialty supervision precedes longitudinal responsibility for primary care patients. This sequencing aims to consolidate diagnostic reasoning, procedural skills, and interprofessional collaboration before trainees assume sustained responsibility in community-based settings. Variations exist across countries; however, the presence of defined supervision and staged responsibility remains a shared principle. Against this background, this narrative review examines the modernization of postgraduate education in general practice to support quality primary care under early clinical responsibility and reform. South Tyrol was selected because it combines early clinical responsibility for general practice trainees, pronounced workforce constraints, and autonomous implementation of Ministerial Decree 77/2022, making it a uniquely informative case to examine how primary care reform interacts with postgraduate training structures in Italy [11,22].

Early clinical responsibility in Italy lacks systematic analysis of educational governance, supervision structures, and accountability when trainees assume substantial contractual and clinical duties in community settings. Workforce shortages and curricular arrangements have been described, but not how responsibility is allocated, supervised, and monitored across distributed primary care environments after recent reforms. In South Tyrol, trainees provide extensive frontline primary care under workforce constraints and organizational transformation, yet system-level supervision and governance have not kept pace [11,22]. Ministerial Decree 77/2022 reorganizes care delivery while supervision capacity, educational accountability, and the role of AI tools in regulating early clinical responsibility remain unresolved.

The central research question of this review is whether traditional apprenticeship-based postgraduate general practice training remains fit for purpose under conditions of early clinical responsibility and primary care reform, and how system-based supervision and AI-supported tools can support educational quality and patient safety in this context. Accordingly, three interrelated research questions concerning postgraduate general practice education under early clinical responsibility were analyzed. Drawing on international evidence and the Italian context shaped by DM 77/2022, this review explores (1) early supervised autonomy and competency development, (2) transition from person-dependent to system-based supervision, and (3) AI’s potential contribution to postgraduate education. This review integrates educational and policy perspectives to inform future training strategies aligned with evolving primary care.

This review addresses a mismatch between the early clinical responsibility of general practice trainees and the person-dependent apprenticeship models that still dominate postgraduate training. In Italy, particularly in South Tyrol, trainees now provide substantial clinical service under workforce shortages and primary care reform yet lack corresponding system-level supervision. This creates risks for educational quality, supervision equity, and patient safety. We examine whether traditional training models meet current needs and consider system-based supervision and artificial intelligence (AI)-supported tools as approaches to modernize postgraduate general practice education for contemporary primary care structures. We synthesize international evidence on early clinical responsibility in general practice and position this within contemporary primary care reform. We evaluate limitations of traditional apprenticeship-based training under workforce shortages and early autonomy and propose system-based supervision as an educational response aligned with multidisciplinary primary care settings. We also examine the role of artificial intelligence in supervision and assessment, focusing on governance, accountability, and ethical requirements.

## 2. Methods

This review compiles the current evidence related to education, organization, and policy in postgraduate general practice education. It emphasizes supervision models, the early stages of supervised clinical independence, community-based training environments, and the role of artificial intelligence in primary-care education. A narrative review design was chosen due to the topic’s coverage of diverse sources, including empirical studies, policy documents, and conceptual frameworks. This approach is particularly suitable for addressing the emerging field of postgraduate general practice education, where the evidence is currently fragmented and not amenable to quantitative synthesis. The review particularly considers the Italian context, highlighting regional differences in training organization, the early clinical responsibilities assumed by trainees, and the effects of primary care reforms following DM 77/2022, using South Tyrol as a case study.

A comprehensive literature search was performed using PubMed (National Library of Medicine, National Institutes of Health, Bethesda, MD, USA) and the Consensus AI-powered academic search engine (Consensus Inc., Boston, MA, USA) with no specific time constraints for the publication date. The search utilized terms such as “general practice training,” “family medicine education,” “postgraduate medical education,” “primary care education,” “clinical supervision,” “competency-based education,” “entrustable professional activities,” “early clinical autonomy,” “community health centers,” “primary care reform,” and “artificial intelligence.” Additionally, policy documents and legal texts pertinent to Italian primary care and postgraduate training, including DM 77/2022 and national regulations on general practice training, were examined in conjunction with peer-reviewed literature.

The review drew on approximately 120 sources, including peer-reviewed empirical studies, systematic and narrative reviews, conceptual framework papers, and national and regional policy documents. We included sources addressing postgraduate general practice education, supervision structures, early clinical responsibility, or educational governance in primary care. We applied no formal exclusion criteria based on study design, given the topic’s heterogeneity and emerging nature. Empirical studies, reviews, and framework papers informed the synthesis. Policy documents and legal texts provided context on health system reform and educational models; we interpreted these analytically rather than as empirical evidence. Selection prioritized applicability to general practice and primary care training over methodological hierarchy. Empirical findings are presented separately from normative and policy-oriented interpretations, which are framed as contextual and forward-looking rather than as direct evidence.

This review focused on supervision, competency development, and educational governance in primary care, including systematic reviews, empirical studies, and framework papers. Rather than formal quality scoring, the assessment was centered on relevance and applicability to postgraduate general practice education. The findings were synthesized using an integrative narrative approach that examined the alignment between educational theory, supervision models, and their application in primary care settings. AI-supported educational tools have been cautiously evaluated as supplements to human supervision. The Italy and South Tyrol cases demonstrated how workforce constraints affect training models.

To enhance transparency and rigor, this review followed the principles of the Scale for the Assessment of Narrative Review Articles (SANRA) [23], addressing clarity of aims, literature search description, referencing, evidence synthesis, and logical narrative structure. We used SANRA as a quality framework to guide study selection, structure the synthesis, and ensure balanced consideration of empirical studies, reviews, and policy documents. We ensured rigor through explicit justification of included sources, consistent thematic analysis, cross-referencing of findings, and reflection on contextual and conceptual limitations.

## 3. Results

### 3.1. Traditional Apprenticeship Models in General Practice

Traditionally, postgraduate education in general practice has followed an apprenticeship model with one-to-one supervision by experienced practitioners in a single practice. This approach has proven effective for professional socialization, mentorship, and clinical knowledge transfer in the field. Evidence shows its value for role modeling, patient exposure, and professional identity development during early training [3,16]. Variation in educational quality across apprenticeship-based training sites, dependent on supervisor characteristics was observed. Whether these variations constitute ‘limitations’ depends on the benchmarks applied and the organizational context. A key weakness is the variable educational quality, which depends on the supervisor’s clinical skills, pedagogy, workload, and innovative mindset [1,4]. This variability challenges equity in postgraduate education and complicates the maintenance of competency standards across sites.

Traditional apprenticeship models no longer align with modern primary care delivery, which emphasizes multidisciplinary and team-based care for chronic disease management and prevention. Single-supervisor models limit exposure to interprofessional collaboration, care coordination, and population-oriented approaches that are essential for general practice [12,24]. Consequently, trainees in isolated practices may be underprepared to work in integrated primary care organizations.

Scalability is a structural constraint. Reviews have identified time pressure, competing demands, and insufficient support as key barriers to effective supervision [1,2]. In systems with workforce shortages, these pressures make it difficult to balance clinical and educational responsibilities of the residents. This is notable in Italy, where early clinical responsibility by trainees addresses primary care shortages and regional heterogeneity [7,11].

Resistance to structural reform among the established general practice workforce remains a significant barrier. Evidence suggests that apprenticeship models may perpetuate existing practices and norms, including skepticism toward team-based care, digital health tools, and new structures such as CdCs [4,25]. This traditional model risks reproducing outdated care paradigms rather than adapting to the evolving primary care systems.

Evidence indicates that while apprenticeship-based supervision remains valuable in postgraduate general practice education, it is insufficient alone for future training. Its limitations in terms of consistency, scalability, and adaptability necessitate the development of system-based supervision frameworks that distribute educational responsibility beyond individual trainers and align with modern primary care.

### 3.2. Early Clinical Responsibility and Supervised Autonomy in General Practice Training

Early clinical responsibility has become central to postgraduate training in general practice, where workforce shortages require trainee integration into service provision. This supervised autonomy places trainees in clinical roles with graduated responsibilities while caring for patients during training. The educational impact of this approach has been studied across various settings, particularly in general practice.

Early responsibility is central to the formation of a professional identity and the development of clinical judgment. In residency training, autonomy fosters ownership and commitment to patient care, which are core attributes of competent practitioners [15]. Responsibility drives learning when it is embedded within supervision and trust frameworks. Workplace-based training programs have shown that supervised autonomy accelerates clinical competence when clear entrustment processes exist. Studies from residencies have demonstrated that entrustable professional activities (EPAs) and progressive supervision relaxation improve performance while maintaining quality [26,27]. In general practice, mutual trust between the trainer and trainee determines autonomy allocation [16].

Research shows that community-based education enhances early responsibility in primary care settings. Reviews indicate that community placements improve decision-making, communication, and teamwork when learners work within care teams [28]. Studies suggest that community-oriented training models lead to equal or better quality of care and preventive practices compared to traditional curricula [17].

In Italy, early clinical responsibility during postgraduate general practice training emerged as a response to primary-care workforce shortages rather than pedagogical reform. Regulations allow trainees to conduct compensated clinical activities under provisional arrangements, thereby enabling early patient care involvement. Implementation varies across regions, based on organizational capacity and local priorities. In South Tyrol, trainees may assume responsibility for patient lists during postgraduate training, typically after initial hospital-based rotations. Supervision is formally organized through designated tutors; however, in practice it is predominantly retrospective or remote, and its availability and intensity may vary across settings. This arrangement illustrates both the potential benefits and risks of service-driven training when clinical responsibility is not embedded in structured on-site supervision frameworks [4,25].

Early autonomy in primary care training can be problematic due to informal or inconsistent supervision. Reviews across low-, middle-, and high-income settings show that unsupported autonomy can lead to stress and uneven learning outcomes, especially in resource-constrained environments [2,29]. However, when early responsibility occurs within team-based care models with reliable referral pathways and feedback mechanisms, it can enhance education and care quality.

The evidence indicates that early clinical responsibility in general practice training is neither inherently harmful nor beneficial; its value depends on structured supervision and organizational support.

Table 1 summarizes the key characteristics distinguishing traditional apprenticeship-based postgraduate training models from emerging approaches characterized by early clinical responsibility and system-based supervision.

### 3.3. Community Centers and Primary Care Reform After Ministerial Decree 77/2022 in Italy: Implications for Postgraduate Training

DM 77/2022 reorients Italian primary care from a fragmented to an integrated, community-based delivery. The key elements include CdCs, expanded services, multidisciplinary teams, and digital infrastructure [7].

CdCs host multiple healthcare professionals, including general practitioners, nurses, social workers, and allied health professionals, within shared organizational structures. These settings differ from traditional, single-practice environments. Evidence shows that training in multidisciplinary primary care organizations improves learners’ exposure to team-based care, care coordination, and population-oriented approaches, which are core competencies for modern general practice [12,13,24]. These competencies are difficult to acquire in isolated apprenticeship models but align with the objectives of DM 77/2022.

Studies have shown that trainees in community-based primary care settings report higher readiness for practice, confidence with complex patients, and understanding of system-level care [14]. However, these benefits require a structured educational governance. Without explicit educational roles, trainees’ risk being absorbed into clinical work without adequate supervision, which compromises the quality of their training.

The Italian reform allows regional autonomy in implementation, creating heterogeneity in CdC organization and training. Some regions integrate these centers into structured training with defined supervision, whereas others operate them mainly as service facilities. This variation reflects regional differences in general practice training capacity, raising concerns about the quality of education across regions [7].

South Tyrol provides an illustrative case. Within autonomous health governance and postgraduate training in general practice, policy discussions foresee trainee clinical activity in CdCs as part of primary care reform. However, the integration of CdCs into postgraduate education remains undefined and these settings are not embedded as training sites within the current curriculum. CdCs do not provide core features of general practice training such as continuity, stable supervision, and cumulative patient knowledge. However, well-governed trainee involvement in CdCs could support competencies increasingly relevant to future primary care, including interprofessional collaboration and care coordination.

The South Tyrolean case underscores the need to align planned service requirements with educational objectives. Without explicit learning goals and supervision, such arrangements risk undermining training quality; with appropriate governance, they may complement practice-based training rather than replace it [11].

The educational value of CdCs requires clear role definitions, supervision models and accountability structures. Reviews of primary care training emphasize designated leadership, protected supervision time and feedback mechanisms [24,30]. Without these elements, CdCs may replicate the limitations of traditional apprenticeships.

Evidence indicates that multidisciplinary, integrated primary care organizations can provide favorable environments for postgraduate general practice education. However, CdCs as currently implemented in several regions, including South Tyrol, often lack the structural characteristics of integrated general practice units and therefore do not inherently function as suitable training sites. However, their educational potential requires explicit recognition as learning organizations, with supervision integrated into the service delivery. Without system-level planning, the expansion of community-based care may exacerbate tensions between workforce needs and educational goals.

Community care hubs function as training environments only when they meet specific criteria: general practitioners with assigned supervisory responsibility; protected time for supervision, feedback, and reflection; learning objectives aligned with general practice curricula; continuity of patient care for longitudinal learning; competency-based supervision frameworks such as EPAs to regulate autonomy; and organizational accountability ensuring educational quality alongside service provision. Without these criteria, community care hubs serve primarily as service sites and cannot function as core training locations.

### 3.4. From Person-Dependent Apprenticeships to System-Based Supervision Models

System-based supervision distributes training responsibility across organizations rather than individual supervisors [31]. Supervisory roles are shared across teams using competency-based frameworks such as EPAs, with standardized assessment, feedback, and quality monitoring. Organizations provide supervision capacity through protected supervisory time, supervisor training in competency assessment, and escalation pathways when needed. Implementation requires coordinated oversight across training sites, documentation of entrustment decisions, and digital tools supporting feedback and continuity. Physicians retain clinical responsibility.

Studies document variable educational outcomes in person-dependent apprenticeship models under conditions of workforce shortage [1,4]. System-based supervision models show improved consistency in feedback provision and assessment standardization across training sites [3,31]. Early clinical responsibility and complex care environments require supervision models that are resilient to workforce and organizational variability. The literature advocates system-based supervision frameworks, where training responsibility is distributed across organizational structures rather than individual supervisors. System-based supervision embeds supervision within care delivery through governance, accountability and standardized processes. Supervision is provided by multiple professionals, including physicians, allied health staff, and educational leaders. Reviews show that these distributed models reduce training quality variability, improve feedback continuity, and enhance scalability under service pressure [1,3,4].

EPAs are key elements of system-based supervision. EPAs translate competencies into defined clinical tasks that can be entrusted to trainees after they have demonstrated competence. By linking responsibility to performance and supervision levels, EPAs provide a framework for managing autonomy while maintaining patient safety [31,32]. Evidence shows that EPA-based frameworks improve supervision transparency and support progression toward independent practice [26,27]. In postgraduate general practice, entrustment criteria apply to activities such as managing patients with chronic multimorbidity, triaging undifferentiated symptoms, coordinating care with community and social services, and making out-of-hours primary care decisions.

System-based supervision models facilitate learning in multidisciplinary primary care settings. Studies have shown that team-based supervision in community clinics enhances collaborative decision-making and care coordination [24,30]. These models ensure that clinical responsibility is distributed across care teams rather than isolated within individual practices, preventing unsupervised service provision.

The relevance of system-based supervision has increased in Italy following DM 77/2022, which promotes CdCs as hubs for multidisciplinary primary care. However, their educational benefits require the explicit recognition of training functions through defined supervisory roles and accountability mechanisms to avoid increasing service pressure on trainees [7]. South Tyrol demonstrates the implications of this transition. The province’s integration of clinical responsibility with training pathways demonstrates the need for supervision beyond individual tutors. System-based supervision through coordinated oversight and curricular alignment enables trainees to benefit from early responsibility, whereas informal supervision magnifies autonomy risks [11].

System-based supervision requires the formal designation of responsibilities, supervisor training in competency assessment, workload monitoring, and institutional support to balance service with education. While maintaining mentorship value, it embeds these relationships within an organizational framework that supports consistency and sustainability. The shift from apprenticeship to system-based supervision aligns with modern primary care, enables structured, supervised autonomy, and maintains educational quality despite service pressures.

Table 2 summarizes the key domains and representative evidence supporting the effectiveness and scalability of system-based supervision models in postgraduate general practice training.

### 3.5. The Role of Artificial Intelligence in Supporting Supervision and Competency-Based Assessment in General Practice Training

As postgraduate education in general practice moves toward system-based supervision and competency frameworks, AI is being explored as a supportive technology to address supervision constraints and feedback needs [34,35,36], particularly in primary care settings with high service demands and workforce shortages. AI tools in postgraduate medical education fall into three categories [33]. Clinical decision-support systems provide alerts, guideline suggestions, and pattern recognition. In primary care education, these work well for narrow tasks but require supervision due to bias and overreliance risks. Learning analytics and documentation tools aggregate clinical data, monitor case mix, and identify learning needs; these increasingly support feedback and reflective learning. Assessment and simulation tools, including AI-assisted feedback on clinical reasoning, communication, and documentation, show promise but lack validation for high-stakes assessment.

AI supports structured educational tasks: documentation review, feedback generation, learning analytics, and simulation-based assessment [37,38,39]. AI cannot perform clinical supervision because supervision requires contextual judgment, ethical responsibility, and legal accountability—capacities AI systems lack and have not been validated for in primary care settings [39,40]. Clinical supervision and professional accountability require physician oversight [41].

AI systems may outperform human experts in narrowly defined diagnostic tasks using large, structured datasets, such as imaging or signal-based classification [39,42]. In postgraduate general practice training, AI cannot replace physician oversight in clinical decision-making, entrustment, or accountability [37,40,41].

AI tools have been implemented across postgraduate medical education, including clinical documentation analysis, simulation training, learning analytics, and feedback systems. Available studies suggest that AI-supported tools may facilitate assessment processes, feedback delivery, and self-directed learning within competency-based curricula, although empirical evidence in postgraduate general practice training remains limited [19,33].

AI shows promise in supporting competency-based assessment frameworks and EPAs through the analysis of clinical encounters and documentation patterns to generate objective indicators of trainee performance and identify supervision needs. AI-driven assessment tools can improve evaluation consistency, although their validity depends on data quality and defined competencies [20,21].

AI facilitates supervision through structured feedback and reflective practice by analyzing clinical notes and consultations to assess the documentation, reasoning, and communication. Studies have shown that trainees value AI-supported feedback as a complement to human supervision [19], particularly in community-based settings where direct observation is limited.

Despite these benefits, the literature highlights the key limitations of AI-supported supervision, including data privacy, algorithmic bias, transparency, and accountability concerns. Reviews emphasize that AI systems should function as decision-support tools within governed frameworks and not as autonomous evaluators [20,21]. Without proper governance, AI tools risk reinforcing inequities and shifting supervisor accountability. Educational settings require safeguards ensuring fairness and transparency, including trainees’ rights to explanation and contestation of AI-assisted assessments. Supervising physicians and training institutions hold legal responsibility for educational and clinical decisions. Governance frameworks must specify data stewardship, validation standards, and human oversight.

In the Italian primary care reform under DM 77/2022, AI-supported supervision may benefit distributed care models in which traditional supervision is challenging. While AI tools can help maintain educational quality across settings through documentation review and monitoring, evidence shows that technology cannot substitute for clear supervisory roles or institutional educational commitment [7]. South Tyrol’s experience further illustrates this balance between tourism and sustainability. The combination of early clinical responsibility, multilingual practice environments, and geographically dispersed training sites amplifies the need for standardized and flexible supervisory support. AI-assisted educational tools have the potential to support more consistent supervision and assessment across distributed training sites, particularly where direct supervision is limited, but their effectiveness in postgraduate general practice has not yet been systematically evaluated [11].

AI can enhance postgraduate general practice education by supporting assessment, feedback, and supervision within system-based frameworks. Its effectiveness requires integration into educational governance, alignment with curricular objectives, and an understanding that AI complements rather than replaces human supervision.

## 4. Discussion

This review examined the evidence on postgraduate education in general practice regarding clinical responsibility, primary care reform, and supervision models. The Discussion addresses each of the three outlined research questions by synthesizing the reviewed evidence across supervision models, organizational settings, and the role of AI. The findings strongly suggest that traditional apprenticeship models are insufficient to meet modern primary care demands. The convergence of supervised autonomy, multidisciplinary care, and competency frameworks requires system-based supervision models, with AI as a supportive educational tool.

The literature indicates that the educational value of early clinical responsibility depends on governance, supervision, and organizational structure. Evidence shows that autonomy enhances professional identity and competence when supported by entrustment processes, supervision, and team support. However, responsibility driven by service needs without formal supervision risks compromising educational outcomes and trainee’s well-being.

Early clinical responsibility without adequate support creates specific risks: inconsistent supervision, reduced reflective learning opportunities, increased trainee stress, and threats to patient safety. These risks increase when responsibility is driven by service needs rather than educational design. Evidence from community-based and resource-constrained environments shows that unsupported autonomy can lead to defensive practice, uneven competency development, and deteriorating supervision quality [2,4,15,25]. Early responsibility requires structured supervision, clear entrustment criteria, and organizational accountability to produce educational benefits.

**Figure 1:**
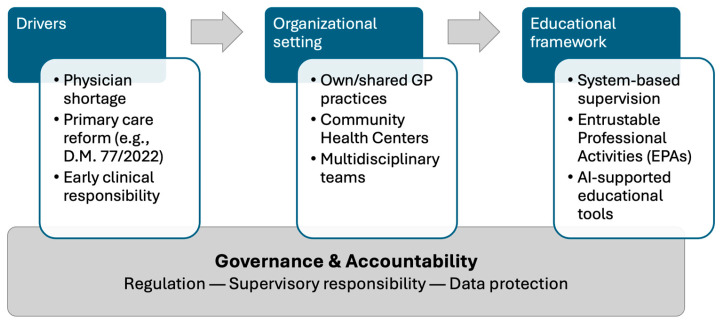
Conceptual framework of future-oriented postgraduate education in general practice under primary care reform. Structural drivers, such as workforce shortages, health system reform, and early clinical responsibility, shape emerging organizational settings, including Community Centers and distributed training sites. These environments require system-based educational frameworks grounded in competency-based supervision and supported by digital and AI-assisted tools. Robust governance and accountability structures underpin all levels of the framework and are essential for aligning service delivery with educational quality. Abbreviations: D.M., ministerial decree (decreto ministerale); GP, general practitioner; EPAs, entrustable professional activities.

To facilitate the interpretation of these findings, Figure 1 summarizes the conceptual framework linking structural drivers, organizational settings, educational models, and governance requirements in postgraduate general practice training.

The Italian context illustrates this tension particularly well. Despite a comparatively high overall physician density, persistent shortages in the primary care workforce and pronounced regional heterogeneity have led to the early integration of trainees into clinical services. DM 77/2022 further amplifies this dynamic by reorganizing primary care delivery around CdCs and multidisciplinary teams; however, current implementation patterns suggest that general practitioners under contract frequently do not participate in these settings, with clinical activity in CdCs increasingly delegated to trainees and early career physicians.

In regional implementations, including South Tyrol, early clinical responsibility is legally framed as the full contractual accountability of the trainee, with supervision primarily organized through retrospective and remote tutoring structures rather than continuous on-site supervision.

Structural decoupling exists between service provision and educational supervision. Trainees deliver primary care in CdCs, while supervision remains in general practice settings. CdCs’ multidisciplinary teams often lack GP specialists, limiting context-specific supervision. This setup weakens direct observation and accountability, shifting supervision toward indirect methods of supervision.

Although primary care reform under DM 77/2022 creates conditions for multidisciplinary collaboration, these settings cannot function as learning environments by default. Without requirements for GP participation, supervisory responsibility, and protected educational time, CdCs may become service-oriented structures that rely heavily on trainees. Service provision may expand without educational governance, intensifying the tension between workforce needs and the quality of training.

**Figure 2:**
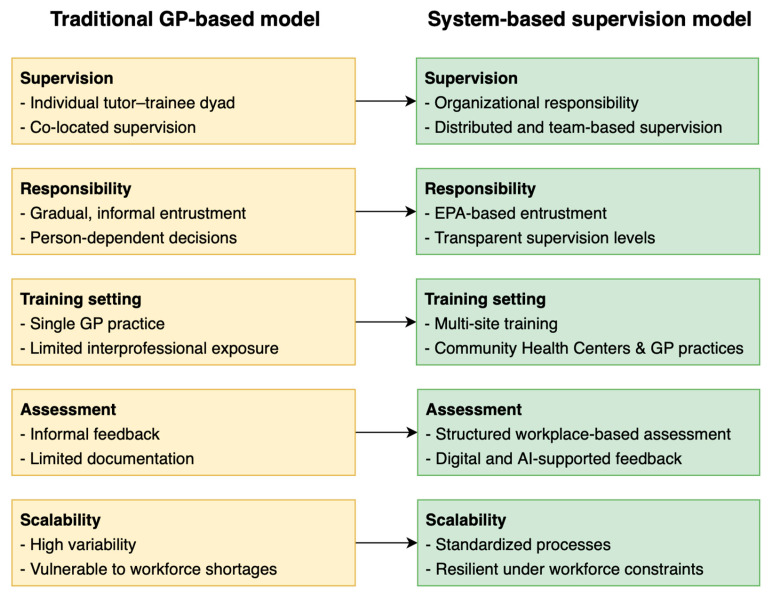
Transitioning from traditional apprenticeship to system-based supervision in postgraduate general practice training. Traditional one-to-one apprenticeship models rely on individual tutors, informal trust, and co-located supervision. In contrast, system-based supervision distributes responsibility at the organizational level, integrates competency-based frameworks such as entrustable professional activities, and supports supervision through digital and AI-assisted tools, thereby improving scalability and consistency under workforce constraints. Abbreviations: AI, artificial intelligence; GP, general practitioner; EPAs, entrustable professional activities.

The contrast between traditional apprenticeship models and emerging system-based supervision approaches in postgraduate general practice is illustrated in Figure 2 below.

System-based supervision is required to address these challenges. Distributed supervision models, based on organizational responsibility rather than individual trainer-trainee relationships, reduce training variability and improve scalability under workforce constraints. EPAs operationalize competency-based education by enabling transparent decisions regarding supervision across training settings. System-based supervision complements longitudinal mentorship within a framework that promotes consistency and resilience.

Training quality depends on organizational culture and team practices, not just supervision structures [43]. Cultures with shared educational responsibility, psychological safety, and openness to feedback support effective supervision, reflective learning, and progressive entrustment. Hierarchical or service-dominated cultures can undermine supervision despite formal structures. Team-based primary care environments enhance learning through distributed supervision, interprofessional feedback, and collaborative decision-making. Fragmented teams and unclear roles reduce training coherence and accountability. Large-scale analyses of team-based online medical consultation platforms show that defined team roles and leadership participation enhance coordination and perceived effectiveness in digital care. Organizational and team structures matter when clinical and educational work is distributed [44].

Artificial intelligence can enable supervision frameworks when human oversight and educational governance are defined. AI tools may enhance feedback, documentation review, analytics, and assessment consistency, where direct supervision is fragmented. However, AI cannot replace professional judgment, ethics, or the relational aspects of supervision. Its value depends on its integration into supervision systems with clear accountability and safeguards against bias and misuse.

The presented evidence indicates that integrated, GP-led group practices and primary care units offer structurally favorable conditions for postgraduate training. These settings preserve longitudinal doctor–patient relationships, enable cumulative patient knowledge, and allow continuous on-site supervision while supporting early, supervised responsibility. Such organizational forms align more closely with the core principles of general practice than service-oriented, episodic care structures.

The literature published in 2024–2025 shows little empirical research on system-based supervision, early supervised autonomy, competency-based frameworks, or AI-supported supervision in postgraduate general practice. Recent studies address these themes primarily in hospital-based specialties, nursing education, or through conceptual discussion of primary care reform and digital education [38,39,40,45,46,47]. While these studies offer useful insights, they do not directly examine postgraduate general practice training. This gap indicates continued need for empirical research in this domain.

Several limitations apply. As a narrative review, this study does not follow systematic review methodology and cannot provide complete or quantitative synthesis of evidence. The analysis draws on heterogeneous literature from different countries, disciplines, and training systems, limiting direct comparability. Empirical evidence specific to postgraduate general practice—particularly on system-based supervision, early supervised autonomy, and AI-supported supervision—is sparse, requiring reliance on indirect evidence from related fields. The Italian and South Tyrolean contexts serve as illustrative cases and may not generalize to other healthcare systems. In addition, narrative reviews risk selection and interpretation bias because study inclusion and evidence synthesis rely on expert judgment rather than quantitative thresholds. We followed SANRA principles, emphasized transparency in source selection, and distinguished empirical evidence from normative and policy interpretation.

### Policy Implications

From a policy perspective, this review has implications for health system reform and postgraduate medical education in Italy and other similar settings.

First, education should be integrated into primary care reforms. While reforms such as D.M. 77/2022 reshape training environments, educational objectives remain secondary. Recognizing CdCs as training sites requires explicit educational responsibility, supervisory roles, and protected training time to maintain quality. Given the absence of evidence demonstrating educational effectiveness of service-oriented CdCs as training sites, and documented risks of inadequate supervision in community settings, policy consideration is needed regarding their designation as primary training locations; if involved at all, their educational role would need to be limited to clearly defined learning objectives that cannot be addressed within GP-led training environments.

Second, early clinical responsibilities should be regulated through competency-based frameworks rather than informal delegation. Authorities should promote EPAs and standardized supervision levels to ensure patient safety and educational equity. Competency-based frameworks such as EPAs offer a transparent and defensible mechanism for linking clinical responsibility to demonstrated competence. In reform contexts, EPAs may serve not only educational but also regulatory functions, providing policymakers with objective criteria for determining when and under which conditions trainees may assume independent clinical duties.

Third, supervision capacity should be viewed as a system resource rather than an individual burden. Policies relying on goodwill or the voluntary participation of practitioners are unsustainable under current conditions. Supervisory responsibility must be institutionally anchored, with investments in training, protected time, and structures to align service delivery with educational goals.

Fourth, the use of AI in postgraduate education requires proactive governance. Policymakers should support the pilot implementation of AI-assisted tools while establishing ethical, legal, and professional standards. Without governance, innovation risks being underutilized or misapplied, undermining the trust and accountability.

Finally, regional autonomy must be balanced with national coherence. Although regional adaptation allows training models to address local needs, it increases the risk of unequal conditions. National frameworks should define the minimum standards for supervision and governance within which regional innovation can occur.

## 5. Conclusions

Postgraduate general practice training is shaped by early clinical responsibility, workforce constraints, and primary care reform. Traditional, person-dependent apprenticeship models have pedagogical value but cannot stand alone in contemporary, multidisciplinary primary care systems. Early supervised autonomy supports competence development and professional identity formation when embedded in governed supervision frameworks. Service-driven responsibility without adequate supervision undermines educational quality, patient safety, and trainee well-being.

System-based supervision is required for modern postgraduate general practice education. Organizationally anchored supervision models using competency-based frameworks such as EPAs enable consistent supervision, transparent entrustment, and scalability across training environments. AI may complement these systems through feedback, assessment, and learning analytics, but requires human oversight and governance.

Aligning service reform with educational governance is essential to sustain training quality. CdCs and reformed primary care settings function as training environments only when clear supervisory responsibility, protected educational time, and organizational accountability exist. Without this alignment, primary care reforms intensify tensions between workforce demands and postgraduate training rather than securing general practice capacity.

## Figures and Tables

**Table 1:** Comparison of traditional apprenticeship-based and emerging system-based postgraduate general practice training models.

Dimension	Traditional GP Training	Emerging GP Training ^1^
Timing of responsibility	Late	Early
Supervision	Individual tutor	System-based
Training setting	Single practice	Multi-site (CdC and GP practice)
Assessment	Informal	EPA-based
Digital support	Minimal	Structured/AI-assisted

^1^ Emerging GP training refers to internationally observed models characterized by early supervised clinical responsibility, distributed training across multiple sites, competency-based assessment (e.g., EPAs), and system-based supervision, as described in European, UK, Canadian, and Australian primary care training literature. Abbreviations: GP, general practitioner; CdC, community center; EPAs, entrustable professional activities; AI, artificial intelligence.

**Table 2:** Key domains and representative evidence supporting system-based supervision in postgraduate general practice training.

Domain	Key Findings	Representative References
Early responsibility	Beneficial if supervised	Grut, 2023; Lyons, 2019 [24,25]
EPAs	Improve transparency	ten Cate, 2015 [31]
Distributed supervision	Scalable, equitable	Kilminster, 2007 [3]
AI in education	Augmentative role	Chan, 2019; Stamer, 2022 [19,33]

Abbreviations: EPAs, entrustable professional activities; AI, artificial intelligence.

## Data Availability

No new data were generated.

## Abbreviations

The following abbreviations are used in this manuscript:

AI	Artificial intelligence
CdC	Community center
DM	Ministerial decree (decreto ministeriale)
EPAs	Entrustable professional activities
SANRA	Scale for the Assessment of Narrative Review Articles
